# THE ASSOCIATION BETWEEN JOB COMPLEXITY AND COGNITIVE FUNCTION IN LATER LIFE ACROSS RACIAL/ETHNIC GROUP

**DOI:** 10.1093/geroni/igad104.0437

**Published:** 2023-12-21

**Authors:** Katy (Qiuchang) Cao, Dawn Carr, Rebekah Carpenter

**Affiliations:** Florida State University, Tallahassee, Florida, United States; Florida State University, Tallahassee, Florida, United States; Florida State University, Tallahassee, Florida, United States

## Abstract

The health of older Black and Hispanic populations are disproportionately impacted by cognitive decline later in life. Although cognitive stimulation through work protects against cognitive decline and dementia in later life, Black and Hispanic individuals have less access to cognitively stimulating employment opportunities compared with their white counterparts. This study aims to disentangle the relationship between work complexity and cognitive function later in life across racial/ethnic groups. Using nationally representative data from the Occupation Information Network (O*NET) linked to the Health and Retirement Study’ Life History Mail Survey (n=1080), we examine how cumulative exposure to cognitively complex work between ages 25 to 55 is associated with cognitive function at age 70 in regressions controlling for cognitive function at age 55 and relevant socio-demographic measures. A composite measure of work complexity was constructed from making decisions and solving problems, thinking creatively, coaching and developing others, frequency of decision making, and freedom to make decisions. Results from regression analyses suggest that compared with cumulative exposure to less cognitively complex work, cumulative exposure to moderately cognitively complex work was associated with a 0.74 point increase in the cognitive score (p<0.01), and cumulative exposure to highly cognitively complex work was associated with a 1.25 point increase (p<0.01) in cognitive score. Among those who held moderately cognitively complex jobs, Black individuals experienced fewer cognitive benefits than their white counterparts later in life. Findings have implications for research and interventions addressing disparities in cognitive health among Black and Hispanic populations through employment-related policies and interventions.

